# Goal-setting to Promote a Healthier Lifestyle in Later Life: Qualitative Evaluation of the AgeWell Trial

**DOI:** 10.1080/07317115.2017.1416509

**Published:** 2018-01-08

**Authors:** Sharon M. Nelis, Jeanette M. Thom, Ian Rees Jones, John V. Hindle, Linda Clare

**Affiliations:** a Centre for Research in Ageing and Cognitive Health, College of Life and Environmental Sciences, University of Exeter, Exeter, UK; b PenCLAHRC, Institute of Health Research, University of Exeter Medical School, Exeter, UK; c School of Medical Sciences, University of New South Wales, Syndey, Australia; d Wales Institute of Social and Economic Research, Data and Methods, Cardiff University, Cardiff, UK; e College of Health and Behavioural Sciences, Bangor University, Bangor, UK

**Keywords:** Behavior change, goal-setting, process evaluation

## Abstract

**Objective**: We report a mixed method evaluation of the feasibility and implementation of the AgeWell goal-setting intervention to promote healthy ageing later life.

**Method**: Researcher field notes, goal-setting interview content, and semi-structured interviews with participants were content analysed to review trial implementation and participants’ perspective on the goal-setting and mentoring intervention.

**Results**: 75 people were recruited: 21 in the goal-setting and 22 in the goal-setting with mentoring arms of the intervention. Goal-setting was feasible in the main domains of interest. Adherence to the protocol was good and the mentoring schedule was adhered to. Participants reported satisfaction with their goal attainment, but barriers for non-achievement were also identified. Recommendations for small changes to the intervention included reducing the number of goals.

**Conclusions**: Participants understood the goal-setting process, and were able to set realistic and achievable lifestyle goals. The intervention and the procedures were acceptable but changes in how goal-setting is both introduced and monitored are needed for wider implementation.

**Clinical Implications**: Goal-setting can be a useful process to help people alter their lifestyle to allow them to age more successfully and reduce risk factors associated with dementia.

## Introduction

Increases in life expectancy have raised the number of people aged over 65 and the number and proportion of people at very old ages (World Health Organization, 2011). Healthy ageing and the promotion of strategies to improve the health of older people has become a key policy initiative (World Health Organization, 2012). In the search for prevention strategies to reduce the risk and extent or delay the onset of dementia, age-related cognitive and physical disability, the AgeWell study (Clare et al., 2012, 2015) trialled an innovative goal-setting approach to the promotion of good mental and physical health in people over 50 years of age.

Goal-setting is an important strategy for behavior change and has been effective in changing physical activity and dietary behavior in adults (Cullen, Baranowski, & Smith, 2001; Shilts, Horowitz, & Townsend, 2004). In the AgeWell study we developed and implemented a naturalistic lifestyle intervention and evaluated its effectiveness among over 50s living in a rural area with limited access to community facilities in a pilot randomized controlled trial (Clare et al., 2012). AgeWell hypothesized that a goal-setting intervention, especially when accompanied by ongoing mentoring, would promote behavior change and optimise engagement, leading to increased cognitive and physical activity, with benefits for cognitive, physical, social and psychological functioning, health and quality of life in people aged over 50. Participants receiving the goal-setting intervention reported higher levels of physical and cognitive activity relative to controls at twelve-month follow-up, and showed improvements on measures of cognition, health, diet and physical fitness (Clare et al., 2015). The intervention was also found to be cost-effective (Jones et al., 2015).

The AgeWell goal-setting intervention, therefore, has potential to be scaled up to benefit larger numbers of people. To optimize generalizability it is important to understand the factors and processes contributing to the intervention effects (Bonell, Oakley, Hargreaves, Strange, & Rees, 2006). In this article we present a process evaluation (Rychetnik, Frommer, Hawe, & Shiell, 2002; Wight & Obasi, 2003) of the AgeWell trial. We review delivery of the intervention by examining fidelity to the intervention protocol. Qualitative interviews will provide a deeper understanding of the feasibility and process of goal-setting. The feasibility and value of mentoring, in addition to goal-setting, will be discussed. Participant reports of the benefits of goal-setting will be explored and the importance of the context within which the intervention was conducted. We consider the overall acceptability of the intervention and recommendations for changes. This article will also draw out implications for further development and future implementation of this lifestyle intervention.

### Study Design

This article describes the process evaluation of the AgeWell trial (Clare et al., 2015). AgeWell was a pilot randomized controlled trial (RCT) of a goal-setting intervention with the primary outcomes of increased cognitive and physical activity and secondary benefits for cognitive, physical, social and psychological functioning, compared to simple discussion of information about activities and health. People completed neuropsychological tests, questionnaires on physical and cognitive activity and well-being. They also completed assessments of physical health, fitness, and a blood sample was taken. Participants were randomly allocated to one of three conditions: information only (C), goal-setting (GS), and goal-setting with mentoring (GSM). The intervention involved a goal-setting interview, with or without follow-up telephone mentoring; participants set up to five goals they wished to work on over the coming year relating to physical activity, cognitive activity, physical health and diet, and social engagement. Participants in the control group had a general discussion about activities and health with the same interviewer. After 12 months all participants reviewed progress with their goals and engage in a semi-structured interview exploring their experiences of and views about the process. All participants benefitted to some extent from attending the Centre, but the participants following the goal-setting benefitted more with improvement on the primary outcomes of physical and cognitive activity, showing that goal-setting offered benefits over and above the general participation. The study was approved by the relevant University and National Health Service Research Ethics Committees.

Throughout the intervention we collected information from researchers and participants to conduct the process evaluation of the AgeWell trial. Quantitative and qualitative data will be used to discuss the implementation of the goal-setting intervention, to examine the experience and opinion of the participants, and provide recommendations for future development of the intervention.

### Participants

The participants were 75 individuals with a mean age of 68.2 years (SD 7.9 years; range from 51–84 years). The majority of the participants were female (86.7%), married (52%), and retired (77.3%). A full description of the sample is provided by Clare and colleagues (2015). Five participants were lost to follow-up, an attrition rate of 6.7%. All participants were interviewed at follow-up and interview data is presented from all 3 arms of the intervention: Control (C) n = 27; Goal-setting (GS) n = 21 (GS); Goal-setting with mentoring (GSM) n = 22.

## Method

### Information Types and Sources

We draw on a number of information types and sources in this process review:

1. Interviewer records and field notes: these contain information on contact with participants, and the interviewer’s experience of delivering the intervention.
2. The Bangor Goal-Setting Interview (BGSI; (Clare et al., 2012): this structured interview schedule was used to record participant ratings of performance and satisfaction in specific goals, and to record additional ratings of goal attainment. Information from the BGSI enabled us to examine the goal-setting process in more detail with information on the number and type of goals, specification of goals across different domains, and extent of goal attainment.
3. Qualitative interviews with participants: on completion of the study participants engaged in a semi-structured interview designed to explore a broad range of issues. Participants were asked about changes experienced over the previous 12 months; specific questions about the intervention; their experience of participating in the trial; any benefits noticed; and recommendations for changes to the study. The interview protocol is provided in Table 1.Interviews lasted on average 15 minutes.

**Table 1.** AgeWell interview protocol.

Control Group
Did the experience of being in the study encourage you to reflect on your own behavior and experiences? Has it influenced/changed the way they feel about things?
Did you ever talk about being in the study with anyone? Who? In what context?
Has the process made you more aware of ageing or changes as we get older? How?
Have you noticed any changes in yourself since last year?
What do you put these changes down to?
Has taking part in the project altered the things you choose to do/activities/diet etc.?
If you had the choice of which group to participate in—which would you have chosen and why?
What was best thing about the research? Any unexpected benefits?
Has anyone else benefited?
What was your least favourite thing about the research?
Is there anything you would change about the research?
Goal-Setting
What difference has it made being part of the research? What did it mean to you?
Did the experience of being in the study encourage you to reflect on your own behavior and experiences? How has it influenced/changed the way they feel about things?
Did you ever talk about being in the study with anyone? Who? In what context?
Has the process made you more aware of ageing or changes as we get older? How?
Have you noticed any changes in yourself since last year?
What do you put these changes down to?
Has taking part in the project altered the things you choose to do/activities/diet etc.?
If you had the choice of which group to participate in—which would you have chosen and why?
How easy was it to set goals?
If asked to choose goals now would they differ from what they chose a year ago?
Would the addition of mentoring have helped you to make change? Explain how?
What was best thing about the research? Any unexpected benefits?
Has anyone else benefited?
What was your least favourite thing about the research?
What things would you change about the research?
Goal-Setting with Mentoring Group
What difference has it made being part of the research? What did it mean to you?
Did the experience of being in the study encourage you to reflect on your own behavior and experiences? How has it influenced/changed the way they feel about things?
Did you ever talk about being in the study with anyone? Who? In what context?
Has the process made you more aware of ageing or changes as we get older? How?
Have you noticed any changes in you since last year?
What do you put these changes down to?
Has taking part in the project altered the things you choose to do/activities/diet etc.?
If you had the choice of which group to participate in—which would you have chosen and why?
How easy was it to set goals?
If asked to choose goals now would they differ from what they chose a year ago?
What was your experience of the mentoring? Timing, frequency
Did mentoring help you to make change? Explain how?
What was best thing about the research? Any unexpected benefits? Has anyone else benefited?
What was your least favourite thing about the research?
What things would you change about the research?

### Analytic Strategy

Interviewer records and field notes are used to inform the fidelity of the intervention protocol by examining adherence to the mentoring schedule and barriers to contact with participants. Goal attainment ratings from the BGSI provide an indication of how successful people were in achieving their goals. Descriptive content analysis of the BGSI goals set at baseline was conducted by two researchers to identify recurring categories of goals. Goals were classifiable in the domains of interest, e.g., physical health and further classified within these domains as appropriate. Interview data were analysed using content analysis (Hsieh & Shannon, 2005), and managed using QSR International’s NVivo 9 software. To develop a coding framework 10% of the interviews, including samples from all 3 conditions, were randomly selected and used in the development of the coding scheme and in coder training. The two researchers met in several calibration meetings to decide on codes to enable consensus between coders resulting in the final coding scheme, which included the code name, code definition, examples from the interview, and coding rules. We calculated coder reliability in two ways: code-recoding and inter-coding (Miles & Huberman, 1994) with the recommended minimum of 10% of the overall sample (Lacy & Riffe, 1996). Coder-recoder reliability for coder 1 was 85% agreement (51 agreements; 9 disagreements) and for coder 2 was 85.5% agreement (47 agreements; 8 disagreements). Inter-coder reliability was conducted via ReCal (Freelon, 2010) and the percentage agreement was 82% agreement (87 agreements, 20 disagreements). The coding framework for the interviews allowed for the inclusion of expected and novel issues within the broad process evaluation themes including the feasibility of goal-setting, impact of the intervention including changes in the assessed outcomes and other benefits, implementation issues, and participant experience of the participation in the AgeWell study. Quotations have been anonymized, and participant identification numbers are shown together with details of gender (F = female; M = male) and group allocation.

## Results

### Fidelity to the Intervention Protocol

The intervention design addressed fidelity by ensuring the intervention was the same within the conditions and that a clear protocol for delivery of the intervention was created. The researcher conducting the interview used interview protocols for all conditions and this protocol was strictly adhered to. The researcher was trained in the interviewing protocols. Delivery of the intervention was monitored by the researcher in their field notes and any deviations from the protocol noted. Goal-setting was recorded in the standardized BGSI and evidence from the field notes and ratings on the BGSI show that it was possible to conduct the goal-setting process with all the GS and GSM participants. All participants were able to complete the goal-setting interview and identify goals to work on demonstrating their understanding of the intervention. Quality assurance of the goal-setting process and specification of SMART goals was conducted by the project lead and feedback provided to the interviewer to ensure all goals met the SMART criteria. Fidelity to the program of mentoring was closely monitored. The interviewer attempted to deliver all 5 mentoring calls; however only 18 of the 24 participants allocated to this arm fulfilled all five mentoring sessions. For six people it proved difficult to maintain this mentoring schedule. Four people missed one session and two people were unavailable for two of the mentoring sessions.

### Feasibility of Goal-setting

On entry to the trial participants were encouraged to identify up to 5 goals to work on throughout the following 12 months. The total number of goals set was 137, with a mean of 2.9 goals (SD ± 1.2, range 1–5). All participants in the two goal-setting (GS and GSM) groups were able to identify goals. Five people identified the maximum number of 5 goals, 9 identified 4 goals, 15 identified 3 goals, 12 identified 2, and 7 identified one goal. Setting the full complement of 5 goals was difficult to achieve. At follow-up 100% of people still involved in the trial were able to rate their goal performance and satisfaction.

Goal-setting was encouraged in four specific domains of cognitive activity, physical health and diet, and social engagement as these were related to the main study outcomes. Within each domain we examined and further categorized the identified goals. Table 2 provides a summary of goals identified by participants on entry to the trial, grouped by domain and category, and gives examples of individual goals within each domain. Goals varied in their degree of specificity and detail. Goals relating to physical activity and diet and health were the most frequently endorsed and reflected the desire to improve general physical health and engage in a more active lifestyle. For example, starting a new activity, increasing current levels of activity, changing to a more healthy diet. Goals in the cognitive domain were less frequently nominated and focused on maintaining or improving cognitive functioning, for example by learning new skills. Few participants generated goals in the social domain but some did suggest they would like to increase their involvement in social activities. A small percentage of goals did not fit easily into one of the four goal domains.

**Table 2.** Goal identification by domain and category on entry to the trial.

Goal Domain	Goals (*n*) (Percentage of Total Goals)	Category and Sample Goals
Physical	50	Increase Physical Activity
	(36.5%)	I will attend at least one hour of exercise class per week.
		I will spend half an hour, 3 times a week doing exercise in the home
		Start New Physical Activity
		I will start swimming and go once a week.
		I will find an exercise class that I enjoy and will attend once a week.
		Achieve One Main Outcome
		In 12 months’ time I will have walked up a mountain.
		In 12 months’ time I will be attending a weekly exercise class
Dietary/health	40	Reduce Intake
	(29.2%)	I will reduce weekly butter intake by half.
		I will reduce my portion size by using a smaller plate for my evening meal. Eat More Healthily
		I will eat a salad for lunch with fruit in it twice a week.
		I will replace crisps and biscuits in my diet for healthier options such as fruit, nuts and crispbreads.
		Lower cholesterol
		In 12 months’ time I will have lowered my cholesterol by 2 points by using cholesterol lowering spread and yoghurt drinks.
Cognitive	24	Learning New Skills
	(17.5%)	In 12 months’ time I will be able to find an item for sale on the internet and buy it without help.
		I will learn to play a game on the computer, and play it twice a week.
		Improving Memory
		In 12 months’ time I will be able to name all the people in the line dancing class.
		Reducing Stress
		In 12 months’ time I will be meditating on a regular basis.
		In 12 months’ time I will be having one full day of relaxation each week.
		Tackling Difficult Tasks
		In 12 months’ time I will have read an historical novel.
		Maintaining Status Quo
		In 12 months’ time my cognitive levels will be the same as today.
Cognitive/Physical	23	Learn New Skills
	(2.2%)	In 12 months’ time I will be able to do a quickstep.
		Make Improvements
		In 12 months’ time I will be better at Tai Chi.
Cognitive/Social	6	Increase Independence
	(4.4%)	I will travel to France on my own.
		I will go on holidays.
		Increase Confidence
		I will have gained more confidence by putting myself in situations outside of my comfort zone
Social	7	Get Out More
	(5.1%)	In 12 months’ time I will have been to the cinema or theatre 4 times.
		In 12 months’ time I will have been to see a show in a theatre.
		Start New Social Activity
		I will join the choir.
		I will join an activity class and attend regularly.
Mixed physical	3	Maintain Status Quo
and diet & health	(2.2%)	To score the same on the physical and psychological tests this time next year.
Other	3	Planning Ahead
	(2.2%)	In 12 months’ time I will have made arrangements for after my death.
		Financial
		I will save up and buy a replacement wedding ring.

Participants’ experiences of negotiating and setting goals were elicited in the interviews. When reviewing the initial establishment and negotiation of goals on entry to the trial most people found the goal-setting process straightforward: “*It was quite easy. It was obvious what I needed to do (137F GS)”;* “*It’s easy to set them. It’s hard to keep them (108F GSM)”* and *“Pretty easy, I could come up with quite a few (115F GS).”* The interviewer’s facilitation of the goal-setting process was important in helping to identify individual goals. Goals adhered to the specific, measurable, achievable, realistic/relevant and timed (SMART) principles (Bovend’Eerdt, Botell, & Wade, 2009). Guidance and prompts provided by the interviewer aided this process: “*We did it together really didn’t we, so yes, it was fine” (173F GS)*. However, a few people found the idea of setting goals more challenging: “*Difficult, because it involved stepping out of my comfort zone (127F GSM),”* and there were a small number who, in retrospect, would have set different or more demanding goals. At follow-up we asked the control group participants if they thought they would have benefited from goal-setting. A small number said that they were motivated to set their own goals, and did not need a formal goal-setting process: “*I think I’m fairly well self-motivated, but I fine-tuned some of them (125M C)”* and *“I’m too independent, really. I’m quite capable of setting goals for myself, you see (120F C).”*

The process of goal-setting enhanced motivation, and the commitment to work on the goals encouraged individual effort: “*Having determined I was going to do something, and somebody else knew about it… my self-esteem wouldn’t let me not do it, it does make you stick at something. (128F GS)”; “Having the goals probably was something to aim for, that somebody was going to ask me about in a year’s time (115F GS),”* and “*I quite like having a bit of a challenge. Something to aim for that’s quite important (176F GS).”* Having experienced the goal-setting process as part of the trial, there were signs that participants would continue to use this approach: “*I think I will have the strength now to do it without anybody prodding me (155F GSM).”* These reports mirror the satisfaction with goal performance ratings that were shown to significantly improve post intervention (Clare et al., 2015).

### Feasibility of Adding Mentoring to Goal-Setting

Participants in the goal-setting with mentoring arm of the trial received mentoring phone calls at the 2,4,6,8,10 month points over the 12-month interval from baseline to follow-up. The mentoring phone calls were an opportunity to discuss progress with individual goals and to provide advice on how to plan to succeed. The mentoring was positively received and motivated people to work on the goals “*It gives you an incentive being spurred on… I wouldn’t have stuck to the diet and walking without it (108F GSM)”;* “*It reminded me that someone was checking on me so I had to keep it up (132F GSM),”* and *“I think it’s good that you keep on top of us otherwise you’d go back to the bad ways (117F GSM).”* People valued this contact and occasion to discuss barriers and reinforce the goals set *“It made me feel like someone cared about me (127F GSM).”* At follow-up when we asked people who had not received mentoring calls if they would have benefitted from this approach there was a mixed response to this suggestion. Some felt that they would find it stressful or unnecessary: “*I think I would have felt pressurized if I’d had phone calls (112F GS)”* and “*No I don’t think it would have made a difference because I am determined. If I want to do it I will do it on my own (101F GS).”*

### Benefits of Goal-setting

We previously reported significant changes in goal performance and satisfaction from baseline to follow-up with positive gains (Clare et al., 2015). Percentage goal attainment was also recorded; 39 goals (28.5%) were fully achieved, and a further 41 (29.9%) met criteria for 50% or 75% attainment. The mean percentage goal attainment in each of the key domains were as follows: Diet/health 68%; Cognitive 50%, Physical 50%, and Social 69%. We also examined the qualitative evidence for goal achievement to illustrate the extent of change over 12 months as reported directly by participants. Corroborating evidence for the improvements in goal attainment ratings was found in participants’ reports of the successful achievement of goals. There was evidence of 100% attainment and a sense of accomplishment: “*It’s made me achieve my goals, I’m very pleased with the results (170F GSM)”;” I’m two stone lighter and I feel much better (133F GSM)”;* “*I’ve lost weight, and I’ve gained strength and mobility in my legs (169M GSM)”; My diabetes is much more controlled. I used to have hypos all the time (116F GS)* and *“I’ve lost four inches on my waist. (169M GSM).”* Some participants felt they had made a significant change or improvement but with an awareness that their goal was partially achieved: “*I’ve lost some weight (137F GS)”* and *“Crosswords for my memory and exercises - I do them but could do more (173F GSM).”* For some the focus was shifted to areas not specified in their original goals: *“Just because I didn’t do them doesn’t mean I haven’t done other things. Except, as I say, maybe choose different things to do to suit me better (133F GSM).”*

A number of perceived barriers to goal attainment and levels of engagement were reported. The impact of health issues on the ability to work on goals was noted: “*I can’t do anything because of this arthritis so I’m no good doing these goals (171F GSM).”* Changes in personal circumstances also negatively influenced goal attainment: *“When this job came—and it threw everything out (154F GS)”* and “*I had such a lot going on in my life—my husband became very ill and I am now his full-time carer (164F GSM).”* Time issues were also cited as a reason for low levels of goal attainment: “*I’m afraid there’s been too much in my life to devote to it that much (109F GSM)”* and “*We were just too busy…it was hopeless (140F GSM).”*

In addition to goal achievement some participants reported other additional or unexpected benefits from taking part in the trial beyond those specified within the individual goals. The process was empowering and people reported taking more charge of their lifestyle: “*Probably for the first time in my life, I’ve looked at my lifestyle; before I’ve taken every day as it came (125M C)”;* “*I feel I’m benefitting simply by being involved (103F C,)” and* “*I feel like I’m taking charge of myself (129F C).”* For others it increased their sense of confidence: “*A feeling of better self-esteem because the things I’d let slip I picked up again and succeeded (128F GS),” and “I did find myself, having—getting some confidence back. (154F GS).”* Participation in the trial also raised awareness of age related issues, and the need to make lifestyle changes: “*It’s made me more conscious of what I’m doing and what I want to do. That time is running out, and if I don’t do things now, I won’t do them (109F GSM)”* and “*It has given me a more positive approach to ageing. Just because I am getting older doesn’t mean I have to give up on trying new things and having new experiences (168F C).”* Participants also noted benefits for their family and friends who were not directly involved, including health benefits: “*My husband has had the same diet as me so he must have benefited (137F GS)”* and improvement in personal relationships: “*We’ve got more to talk about (107F C),”* and “*Because you’re enjoying it, and you’re happy in yourself, the family has felt the benefit (111F GSM).”*

### The AgeWell Context

The trial was conducted in the context of a newly opened Centre for over 50s, called the ‘AgeWell Centre,’ which ran three days per week in a village community building in rural Wales in the United Kingdom. The Centre was set up by the Age Cymru Gwynedd a Mon charity to promote wellbeing and create social gathering opportunities for people in the community. The context of the AgeWell Centre was important in the success of the trial and to participant experience. From a recruitment perspective it helped that everyone who attended the Centre was offered the opportunity to take part in the trial. The Centre offered a base for data collection, although space limitations sometimes made it difficult to arrange private consultations with participants. Goals could be specified in relation to activities taking place within the Centre, but the Centre did not always offer specific activities to support goals, and these needs were met outside the Centre. Participants valued the opportunity to access the Centre, and the choice of activities on offer: “*It’s something to look forward to, it’s made my week a lot better, until this centre opened there was not a lot to join in with (107F C).”* It facilitated also social interaction and this was appreciated: “*Coming here, mixing and making friends and something to get up in the morning for (107F C)”* and *“It’s brought us a lot together (123F GS).”*

### Overall Acceptability of the Trial and the Intervention

The majority of comments indicated that participation was enjoyable and rewarding. Participants chose to take part in the trial for a number of reasons including wider appreciation of lifestyle issues: “*I was glad to be part of research because if people don’t research on things they never learn, do they? (111F GSM),”* and *“It made me aware of physical and mental issues and gave me a chance to rectify them in a different way to one I would have chosen (127F GSM).”* Participation would also benefit others in the future: *“I think anything that we do, if it’s going to improve the people that are coming along behind us, that is very important (110F C).”*

All participants were asked if they had recommendations about ways in which the trial could be changed or improved upon: 55 (79%) of those interviewed at follow-up were satisfied with the procedures and had no recommendations to make for future implementation. Some minor suggestions to alter the protocol included: changing the 12-month time interval: “*Maybe change it to 6 months instead of 12. Just for peace of mind to see the results earlier (108F GSM).”* There were a few concerns about the assessments conducted as some people found the fitness tests challenging and in some instances difficult due to current physical problems: “*The physical tests, I think. I thought those were a bit excessive (118F C).”* Compliance with the request for a blood sample was lower than expected, with twenty people refusing this at follow-up; reasons for refusal included fear of needles: “*It was having the blood taken that’s the only thing—anything else I’m not bothered about, just the blood (114M C).”* Performance on the cognitive tests were a cause of concern for some people, and could be linked to a fear of developing dementia: *“I think that any sort of —any feedback, which would have been negative towards me was a fear, if you like (105M GSM).”*

## Discussion

The present article evaluates the process of the AgeWell goal-setting intervention to promote healthy ageing and reduce risk of dementia in later life. Goal-setting was feasible for the majority of participants and fidelity to the intervention protocol was good. Few identified the maximum target of five goals with the majority opting to choose two to three goals to work on. This has implications for future interventions as it suggest people wish to focus on a small number of specific goals. The majority of goals were focused on physical activity, diet, and health all of which are considered important and modifiable risk factors for dementia (Biessels, 2014). Participants placed less emphasis on goals in the cognitive and social domains. Research evidence highlights the importance of social networks, social engagement (e.g., (Seeman & Crimmins, 2001) and participation in cognitive activities (e.g., (Glass, 1999) for well-being, reducing the risk of dementia, and survival in later life. It may be that health promotion messages have made people aware of the importance of diet and physical activity for ageing successfully, but people may be less aware of the significance of social and cognitive factors. Prior to the implementation of a lifestyle intervention, education on the evidence of dementia risk factors may be required with people to allow them to make informed choices of goals.

Goal negotiation involved establishing goals that were important to the individual person, in areas where changes were needed, and where change was considered possible. Participant feedback on this negotiation phase emphasized the importance of SMART principles in the identification and specification of goals (Bovend’Eerdt et al., 2009). Participant feedback on the negotiation phase emphasized the importance of the co-production of goals with the interviewer. Allowing people to participate in the goal-setting process in this way creates a sense of ownership and personal importance (Locke & Latham, 2002). Despite the adoption of SMART principles as part of the protocol there was some variability in the specificity and level of detail of some goals, and on reflection some people would have chosen harder goals. This negotiation phase is important to get right as evidence suggests that goals that are difficult and goals that are specific result in greater levels of achievement (Locke, 1996).

Participants reported that having identified specific goals that they were highly motivated to work on these. This motivational effect of establishing goals has been described as operating thorough a number of mechanisms including focusing attention and effort on activities relating to the specific goal, and setting goals requires the person to use the knowledge and skills they have or need to acquire to achieve goal attainment (Locke & Latham, 2002). Recognizing that the goals would be monitored through the research study was a strong motivator. This is similar to the observation in goal-setting theory that making a public agreement to the goal enhances commitment as it becomes a matter of integrity for the individual (Locke & Latham, 2002).

For those in the mentoring arm of the intervention the opportunity to discuss progress with goals at regular intervals was welcomed. The mentoring calls allowed for some early feedback on progress, and to discuss barriers to goal achievement. Goal-setting has been shown to be most effective when there is feedback showing progress in relation to the goal (Locke, 1996). There is evidence that people do not monitor their own goal progress—a phenomenon referred to as “the ostrich problem” (Webb, Chang, & Benn, 2013)—and may avoid considering goal progress if the information is not useful or accurate (Chang, Webb, & Benn, 2017). This feedback was considered helpful for some, when others were asked if they would have liked this option they did not view it as an opportunity for feedback but rather felt it could be intrusive and off-putting. In practice offering a range of options may be necessary for future implementation, to account for differing preferences. Mentoring was implemented with some small deviations from the original protocol. There were some difficulties in maintaining contact with participants. Steps to correct this could include providing a schedule of dates at the initial assessment or participant notifications to the interviewer of any absences at the assigned time.

As for the benefits of goal-setting participants reported high levels of achievement in their personal goals with some clear markers of success. Goal-setting theory advances the idea that goals are an outcome to aim for and a standard for judging satisfaction (Locke & Latham, 2002). From the perspective of people in the AgeWell study the successful achievement of goals did create a sense of personal satisfaction. Barriers to goal achievement were also highlighted including health problems and changes in personal circumstances. People underestimated the impact of health conditions on goal progress and in planning interventions it is important to recognize that older people may be living with significant illnesses or develop health issues during the intervention interval. Benefits were reported for extended family members and friends as people taking part in the trial made lifestyle changes that also influenced others.

Participants were satisfied with their involvement in the trial, and the community Centre context within which the intervention was facilitated was considered important. Participation in the study raised awareness of age-related changes and allowed people to consider making changes to their lifestyle they may not have considered otherwise. This raised awareness may be beneficial as awareness of age-related changes may translate into behaviors that may optimize, maintain or compensate for changes as people age (Diehl & Wahl, 2010).

For the purposes of the AgeWell study we specified the goal domains to reflect known risk factors for dementia but the goal-setting approach is adaptable and can include goals of any type and can be tailored to any type of intervention. There are potentially alternative approaches that could be taken to implementing the AgeWell intervention. One interviewer provided the goal-setting interventions but this could be conducted in different contexts outside of the Centre and presented through different media. There is some evidence that goal-setting approaches can be used in groups as part of community-wide health promotion and community development (Kloseck, 2007; Kolip & Schaefer, 2013) and group administration of the AgeWell protocol could be considered. The application of goal-setting interventions could also be been adopted in web-based formats (O’Donnell, Greene, & Blissmer, 2014), although further evidence is required to assess the effectiveness of this type of online administration.

It should be noted that the majority of participants were women, and there were some challenges in recruiting men who were less frequent attendees at the Centre. The Centre was opened to people over 50 years of age and people who took part in the intervention were from both middle to old age. The goal-setting intervention may apply across age groups but testing the intervention specifically with young old, middle old and very old groups may better inform if the intervention is suitable for all older people.

Overall the goal-setting intervention for behavior change was feasible and acceptable in producing lifestyle changes. This process evaluation of the AgeWell intervention shows that participants could understand the concept of goal-setting and were engaged with the process. They were able to set realistic and achievable goals, and demonstrated progress with goal attainment in the domains of physical activity, cognitive activity, physical health and diet, and social engagement. Some participants noted the benefits of the goal-setting approach and found the experience motivational and empowering. The protocol could be easily adapted for group or online administration offering the opportunity to reach greater numbers. The AgeWell goal-setting intervention offers promise for behavior change in mid- to late-life to reduce a range of risk factors for dementia.

## Clinical Implications

- Goal-setting can be a useful process to help people make changes to their lifestyle.
- The goal-setting intervention could help individuals reduce risk factors associated with dementia and to allow people to age more successfully.

## References

[CIT0001] Biessels G. J. (2014). Capitalising on modifiable risk factors for Alzheimer’s disease. *The Lancet Neurology*, 13, 752–753. doi:10.1016/S1474-4422(14)70154-1 25030506

[CIT0002] Bonell C., Oakley A., Hargreaves J., Strange V., & Rees R. (2006). Research methodology: Assessment of generalisability in trials of health interventions: Suggested framework and systematic review. *BMJ: British Medical Journal*, 333, 346–349. doi:10.1136/bmj.333.7563.346 16902217 PMC1539056

[CIT0003] Bovend’Eerdt T. J., Botell R. E., & Wade D. T. (2009). Writing SMART rehabilitation goals and achieving goal attainment scaling: A practical guide. *Clinical Rehabilitation*, 23, 352–361. doi:10.1177/0269215508101741 19237435

[CIT0004] Chang B. P. I., Webb T. L., & Benn Y. (2017). Why do people act like the proverbial ostrich? Investigating the reasons that people provide for not monitoring their goal progress. *Frontiers in Psychology*, 8. doi:10.3389/fpsyg.2017.00152 PMC5297323 28228740

[CIT0005] Clare L., Hindle J. V., Jones I. R., Thom J. M., Nelis S. M., Hounsome B., & Whitaker C. J. (2012). The AgeWell study of behavior change to promote health and wellbeing in later life: Study protocol for a randomized controlled trial. *Trials*, 13, 115. doi:10.1186/1745-6215-13-115 22827885 PMC3433386

[CIT0006] Clare L., Nelis S. M., Jones I. R., Hindle J. V., Thom J. M., Nixon J. A., … Whitaker C. J. (2015). The Agewell trial: A pilot randomised controlled trial of a behaviour change intervention to promote healthy ageing and reduce risk of dementia in later life. *BMC Psychiatry*, 15, 25. doi:10.1186/s12888-015-0402-4 25880911 PMC4337106

[CIT0007] Cullen K. W., Baranowski T., & Smith S. P. (2001). Using goal setting as a strategy for dietary behavior change. *Journal of the American Dietetic Association*, 101, 562–566. doi:10.1016/S0002-8223(01)00140-7 11374350

[CIT0008] Diehl M. K., & Wahl H.-W. (2010). Awareness of age-related change: Examination of a (mostly) unexplored concept. *The Journals of Gerontology Series B: Psychological Sciences and Social Sciences*, 65B, 340–350. doi:10.1093/geronb/gbp110 PMC2853600 20008026

[CIT0009] Freelon D. G. (2010). ReCal: Intercoder reliability calculation as a web service. *International Journal of Internet Science*, 5, 20–33.

[CIT0010] Glass T. A. (1999). Population based study of social and productive activities as predictors of survival among elderly Americans. *British Medical Journal*, 319, 478–483. doi:10.1136/bmj.319.7208.478 10454399 PMC28199

[CIT0011] Hsieh H.-F., & Shannon S. E. (2005). Three approaches to qualitative content analysis. *Qualitative Health Research*, 15, 1277–1288. doi:10.1177/1049732305276687 16204405

[CIT0012] Jones C. L., Edwards R. T., Nelis S. M., Jones I. R., Hindle J. V., Thom J. M., … Clare L. (2015). Cost-effectiveness findings from the Agewell pilot study of behaviour change to promote health and wellbeing in later life. *Health Economics & Outcome Research: Open Access*, 1, 105. doi:10.4172/heor.1000105

[CIT0013] Kloseck M. (2007). The use of goal attainment scaling in a community health promotion initiative with seniors. *BMC Geriatrics*, 7, 16. doi:10.1186/1471-2318-7-16 17605827 PMC1950866

[CIT0014] Kolip P., & Schaefer I. (2013). Goal attainment scaling as a tool to enhance quality in community-based health promotion. *International Journal of Public Health*, 58, 633–636. Kolip2013. doi: 10.1007/s00038-013-0471-4 23619722

[CIT0015] Lacy S., & Riffe D. (1996). Sampling error and selecting intercoder reliability samples for nominal content categories. *Journalism & Mass Communication Quarterly*, 73, 963–973. doi:10.1177/107769909607300414

[CIT0016] Locke E. A. (1996). Motivation through conscious goal setting. *Applied & Preventive Psychology*, 5, 117–124. doi:10.1016/S0962-1849(96)80005-9

[CIT0017] Locke E. A., & Latham G. P. (2002). Building a practically useful theory of goal setting and task motivation - A 35-year odyssey. *American Psychologist*, 57, 705–717. doi:10.1037//0003-066x.57.9.705 12237980

[CIT0018] Miles M. B., & Huberman A. M. (1994). *Qualitative data analysis: An expanded sourcebook*. Thousand Oaks, California: Sage.

[CIT0019] O’Donnell S., Greene G. W., & Blissmer B. (2014). The effect of goal setting on fruit and vegetable consumption and physical activity level in a web-based intervention. *Journal of Nutrition Education and Behavior*, 46, 570–575. doi:10.1016/j.jneb.2014.03.005 24857600

[CIT0020] Rychetnik L., Frommer M., Hawe P., & Shiell A. (2002). Criteria for evaluating evidence on public health interventions. *Journal of Epidemiology and Community Health*, 56, 119–127. doi:10.1136/jech.56.2.119 11812811 PMC1732065

[CIT0021] Seeman T. E., & Crimmins E. (2001). Social environment effects on health and aging: Integrating epidemiologic and demographic approaches and perspectives. *Annals of the New York Academy of Sciences*, 954, 88–117. doi:10.1111/j.1749-6632.2001.tb02749.x 11797869

[CIT0022] Shilts M. K., Horowitz M., & Townsend M. S. (2004). Goal setting as a strategy for dietary and physical activity behavior change: A review of the literature. *American Journal of Health Promotion*, 19, 81–93. doi:10.4278/0890-1171-19.2.81 15559708

[CIT0023] Webb T. L., Chang B. P. I., & Benn Y. (2013). ‘The ostrich problem’: Motivated avoidance or rejection of information about goal progress. *Social and Personality Psychology Compass*, 7, 794–807. doi:10.1111/spc3.12071

[CIT0024] Wight D., & Obasi A. (2003). Unpacking the black box: The importance of process data to explain outcomes In: Stephenson J. M., Imrie J., & Bonell C. (eds), *Effective sexual health interventions: Issues in experimental evaluation* (pp. 151–166). Oxford, UK: Oxford University Press.

[CIT0025] World Health Organization (2011). *Global health and aging*. Geneva, Switzerland, USA: Author Retrieved from http://www.who.int/ageing/publications/global_health.pdf

[CIT0026] World Health Organization (2012). *Policies and priority interventions for healthy ageing*. Copenhagen, Denmark: Author Retrieved from http://www.euro.who.int/__data/assets/pdf_file/0006/161637/WHD-Policies-and-Priority-Interventions-for-Healthy-Ageing.pdfPlease

